# Postictal Mania Versus Postictal Psychosis

**DOI:** 10.7759/cureus.3338

**Published:** 2018-09-19

**Authors:** Sukaina Rizvi, Faiza Farooq, Shanila Shagufta, Ali M Khan, Yasir Masood, Hina Saeed

**Affiliations:** 1 Psychiatry, Kings County Hospital Center, Brooklyn, USA; 2 Psychiatry, Dow Medical College, Karachi, PAK; 3 Psychiatry, Delaware Psychiatric Hospital, Corona, USA; 4 Psychiatry, University of Texas Rio Grande Valley, Harlingen, USA; 5 Psychiatry, Dr. Ziauddin Hospital, Karachi, PAK; 6 Psychiatry, Sindh Medical, Ontario, CAN

**Keywords:** pos-ictal mania, post-ictal psychosis, generalized tonic clonic, postictal mania, postictal psychosis

## Abstract

PIM (postictal mania) or PIP (postictal psychosis) usually comes on after a single episode or a seizure cluster of generalized tonic-clonic or complex partial secondarily generalized seizures. Patients maintain a lucid interval of clear consciousness which precedes a psychotic episode. The symptoms may include insomnia, hallucinations, delusions, elated expansive mood, euphoria, and distractibility. We present a case of a 62-year-old male with PIP or mania preceding an episode of seizure. In the light of this case report, we illustrate the importance of being vigilant about the psychotic symptoms in a patient with epilepsy in order to minimize the morbidity.

## Introduction

Occasionally, patients suffer a psychotic outbreak after undergoing a seizure cluster lasting several days, usually after maintaining lucidity for a short period of time. The state of mental disturbance that comes on after a seizure bout may leave the patient with confusion, dementia, hallucinations, delusions, with irritability leading to violence in rare instances [1,2].

It is estimated that the prevalence of psychosis in patients with epilepsy is approximately 7.8% higher as compared to the general population, with around 7.2% of patients having suffered or currently having a psychotic illness during the course of epilepsy [3]. However, postictal psychosis appears to be much less common with a prevalence of 2% in epilepsy [3]. Psychoses of epilepsy (POE) usually occur in postictal or interictal states but rarely also occur in the ictal state. Interictal psychosis refers to a state of chronic psychosis that is not associated with a seizure occurrence, basically accounting for about 10 to 30% of psychotic outbreaks seen in epilepsy [2]. Ictal psychosis, which is a rare entity, can be defined as the occurrence of psychosis during an ongoing seizure episode. However, the psychotic outbreak after a lucid interval following a seizure is described as postictal psychosis [2].

In this case we emphasize the importance of monitoring patients for psychosis or mania after a seizure cluster and describe the classic features, review the diagnostic criteria, risk factors, and treatment options recommended by the experts.

## Case presentation

All the listed authors of the case report were involved in the care of the patient in the hospital at that time. However, afterwards most of the authors moved forward into residencies/observerships and are currently residents/observers at other facilities.

We herein present a case of a 62-year-old male with a longstanding (more than 20 years, as per the patient) past medical history of hypertension (HTN), diabetes mellitus (DM), hyperlipidemia (HLD), and seizure disorder. The patient was initially brought to the hospital because he was acting bizarre. The patient's family reported that he had an episode of generalized tonic-clonic (GTC) five days ago while he was walking outside. The patient suffered a loss of consciousness (LOC) of unknown duration. He denied any head trauma at that time. The patient was transferred to the hospital for further evaluation. Baseline labs, including complete blood count (CBC), basic metabolic panel (BMP), liver function tests (LFTs), electrocardiogram (ECG), and computed tomography of the brain (CT brain) were all unremarkable and he was discharged the following day as per the patient. As per the family's account, the patient became extremely active after this event and was found to be talking excessively at home, which was unusual for him. The patient endorsed delusions of paranoia that people were chasing him. He also endorsed visual hallucinations in the context of seeing dead family members at home and was reportedly pacing at home. His appetite was significantly impaired and he refused to eat and sleep for the last three days. The patient admitted to having a lot of energy by stating, "I want to record a video on the internet". At the time of his admission, he presented with an elated expansive mood. He demonstrated rapid speech with a flight of ideas. The thought content was disorganized with delusions of grandeur as he stated, "I own this universe". Baselines labs, including CBC, BMP, LFTs, ECG, and CT scan of brain were all unremarkable. Electroencephalogram (EEG) was also ordered and demonstrated a diffuse slowing of the background with no epileptic foci. The patient denied any fevers, chills, headache, weakness, numbness, tingling, neck pain, double vision/vision changes, chest pain, shortness of breath, and palpitations at that time. As per his sister's account, the patient had a prior history of alcohol-induced seizure 10 years ago and one prior episode of postictal mania 10 years ago that lasted for one week. His last seizure, described as GTC was three months ago. The patient was prescribed Dilantin per oral at that time. On our evaluation, the patient was not able to provide us with the dose of his medication. The patient did not follow up with a neurologist and was non-complaint with his anti-epileptic drugs (AEDs). His Dilantin levels were ordered, which later came out to be 5 ug/ml, which was sub-therapeutic. Treatment was started with divalproex delayed-release per oral 500 mg twice daily, olanzapine 5 mg per oral, folic acid, thiamine, and aspirin 81 mg per oral once daily, after which he showed improvement.

## Discussion

The patient in this case report had psychotic symptoms in the presence of clear consciousness for days after an episode of seizure and reportedly with no evidence of extraneous factors including anticonvulsant toxicity, drug withdrawal, and a prior episode of interictal psychosis. However, this particular episode points towards the psychiatric diagnosis of mania with psychotic symptoms according to the criteria given by the International Classification of Diseases (ICD)-10. The clinical picture given here partly resembled earlier reports in the postictal period [4,5].

The prevalence of postictal psychosis (PIP) is only 7% in treatment-resistant patients with epilepsy [6]. While episodes of PIP are usually short-lived, remission may occur over a period of several days ranging from three days to two weeks [7]. Patients with recurrent episodes of epilepsy are at risk of developing chronic psychosis. Psychotic symptoms are often pleomorphic, including hallucinations (visual or auditory), thought content abnormalities such as delusions (somatic, grandiose, religious, paranoid or others) or tangentiality or loose association, thought blockage, disinhibition of sexual behavior, mental diplopia, illusional familiarity, mood disturbances, and pressured speech. However, a minor subset of patients may have co-occurring manic symptoms after an acute episode of seizure cluster [8,9]. PIP constitutes 25% of psychotic episodes in epilepsy but sadly it is still underrepresented in psychiatric literature [5,10]. Furthermore, there are instances where cases of postictal mania (PIM) have been reported but none of the studies have shown the pathology and the clinical features of PIM as a separate entity [6].

The cardinal features of PIM were elated, expansive mood, euphoria, and distraction in thought process throughout the episode of PIM. In addition, there is a predisposition to show pressured speech, decreased sleep, hyperactivity, a flight of ideas, hyper-religiosity, and grandiosity. Hyper-religiosity and grandiosity were present throughout these episodes in congruence with the mood component fulfilling the ICD-10 criteria for manic disorder and mood (affective) disorder, except the etiology. However, patients with episodes of PIP were more likely to show hallucinations (auditory or visual), delusions (persecutory or delusion of reference), and insomnia; the elated mood was shown to be amongst the most striking features of these episodes of PIP. Some patients also manifest euphoria and elated mood but these symptoms were only present for a short duration of time and never throughout these episodes. Moreover, the hallucinations and delusions were not in congruence with the mood component fulfilling the ICD-10 criteria for transient and acute psychotic disorder and schizophrenia-like organic delusional disorder, except the etiology [6,11].

Postictal psychosis poses a threat of profound morbidity and it is crucial to be aware of the known risk factors associated with it. Episodes of PIP are often preceded by insomnia, a seizure cluster, longstanding history of generalized tonic-clonic or secondarily generalized complex partial seizures. Prior history of PIP, psychiatric hospitalization or psychosis, a long history of epilepsy, bilateral seizure foci (esp. temporal), history of encephalitis or traumatic injury of brain, and low intellectual functioning are all known risk factors [5].

Both PIP and PIM had an episode of secondarily generalization and/or complex partial seizures followed by maintenance of a lucid interval for an average period of 1.9 days for PIP and two days for PIM before showing psychotic symptoms. However, it was noted that the total duration of episodes of PIM was comparatively longer than that of PIP and with a greater number of recurrence. The reported ages for onset of seizure disorder were noted to be older for PIM as compared to PIP [11]. Moreover, PIP has shown a strong association with frontal and temporal lobe epilepsies with the epileptogenic zone showing no laterality on the dominant hemisphere whereas PIM is more often seen with the epilepsy of temporal lobe with epileptogenic foci on the dominant side of the language center. On EEG, epileptiform discharges of lower frequency were shown interictally if performed early in both PIP and PIM. In addition, increased perfusion was seen in frontal and/or temporal lobe epilepsy during the acute episodes of both PIP and PIM on single-photon emission computed tomography (SPECT) [11]. According to Nishida T et al., three patients showed an increase in perfusion during an acute episode of PIM on the non-dominant language side or bilaterally whereas the same number of patients going through an episode of PIP showed an increase in perfusion ipsilaterally to epileptogenic zones [11]. Therefore, physicians should be cognizant of these differences between PIP and PIM while evaluating the patients post seizure.

All patients with PIP and PIM are treated with neuroleptics throughout their episodes of postictal psychosis proving again that PIM lasts longer than PIP. Furthermore, there are no reports of psychotic symptoms postictally in patients with PIM according to Nishida T et al. [11]. To minimize the morbidity of PIP, prompt recognition is the key. Most patients with hallucinations and delusions do not report the symptoms spontaneously and, thus, can only be recognized by specific questions. Therefore, administration of antipsychotic and anti-epileptic drugs early would prove to be beneficial [1,12].

## Conclusions

Postictal psychosis or mania poses a considerable challenge to physicians and they often go unrecognized. Although PIP contributes to 25% of psychosis associated with epilepsy, it is still underrepresented. It can be concluded that even though these syndromes are well recognized, standard diagnostic manuals lack the acknowledgment, hence there is a relative neglect in the literature. Therefore, physicians should judiciously look for the symptoms of PIP or PIM in all the patients with known risk factors. Treatment with antipsychotics or anti-epileptics should be considered at the onset of insomnia to abort a psychotic outbreak or at least curtail morbidity.
